# Trachoma: Protective and Pathogenic Ocular Immune Responses to *Chlamydia trachomatis*

**DOI:** 10.1371/journal.pntd.0002020

**Published:** 2013-02-14

**Authors:** Victor H. Hu, Martin J. Holland, Matthew J. Burton

**Affiliations:** 1 Clinical Research Department, Faculty of Infectious and Tropical Diseases, London School of Hygiene and Tropical Medicine, London, United Kingdom; 2 Kilimanjaro Christian Medical Centre, Moshi, Tanzania; University of California San Francisco, United States of America

## Abstract

Trachoma, caused by *Chlamydia trachomatis* (*Ct*), is the leading infectious blinding disease worldwide. Chronic conjunctival inflammation develops in childhood and leads to eyelid scarring and blindness in adulthood. The immune response to *Ct* provides only partial protection against re-infection, which can be frequent. Moreover, the immune response is central to the development of scarring pathology, leading to loss of vision. Here we review the current literature on both protective and pathological immune responses in trachoma. The resolution of *Ct* infection in animal models is IFNγ-dependent, involving Th1 cells, but whether this is the case in human ocular infection still needs to be confirmed. An increasing number of studies indicate that innate immune responses arising from the epithelium and other innate immune cells, along with changes in matrix metalloproteinase activity, are important in the development of tissue damage and scarring. Current trachoma control measures, which are centred on repeated mass antibiotic treatment of populations, are logistically challenging and have the potential to drive antimicrobial resistance. A trachoma vaccine would offer significant advantages. However, limited understanding of the mechanisms of both protective immunity and immunopathology to *Ct* remain barriers to vaccine development.

## Introduction

Trachoma results from infection of the conjunctiva with *Chlamydia trachomatis* (*Ct*) and is the commonest infectious cause of blindness worldwide. The World Health Organization (WHO) estimates that at least 1.3 million people are blind from trachoma and 40 million have active disease; it is also part of the Neglected Tropical Diseases Programme [1], [2]. It is caused by *Ct* serovars A to C and is generally found in poor, rural areas in less developed countries.

Genital tract *Ct* infection, the commonest bacterial sexually transmitted disease worldwide, infecting 90 million people each year, is caused by serovars D to K [3]. The ocular and genital strains are strictly differentiated with ocular strains lacking tryptophan synthase function [4]. Both ocular and genital infections produce inflammatory reactions, which are often asymptomatic, leading to scarring complications with significant morbidity in a subset of those infected. The WHO recommends the SAFE Strategy for trachoma control: Surgery for trichiasis, mass Antibiotic distribution to treat infection, improved Facial hygiene, and Environmental improvements to interrupt *Ct* transmission [5]–[7]. While implementation of this strategy appears to be effective in reducing active ocular infection with *Ct*, successfully carrying it out on a programmatic level can be challenging in the areas where trachoma is found, and the long-term effect on the scarring stages of trachoma is unknown.

The immune response to *Ct* provides incomplete protection and is known to be important in the development of tissue damage and sequelae including blinding complications. However, protective immune responses and disease pathogenesis in trachoma remain poorly understood. The “immunological paradigm” suggested that disease pathology is a result of cell-mediated immune (CMI) responses against specific chlamydial antigens. The “cellular paradigm” states that infected epithelial cell responses drive pathology through the release of various mediators, and this is supported by recent studies in humans and animal models, which have highlighted the importance of an innate immune response in active and scarring trachoma.

This article reviews the literature on immunity and immunopathogenesis in trachoma. Human studies of the pathology, immune response, and immunopathogenesis in trachoma are comprehensively summarised. Whilst focusing on these human studies of ocular *Ct* infection, we also draw on relevant studies of genital tract infection and animal models. Ocular infection of nonhuman primates leads to a self-limiting follicular conjunctivitis after a single innoculum, and chronic inflammation, conjunctival scarring, and trichiasis with repeated inoculations [8]–[12]. Such a response is similar to that seen in humans, and this model has useful parallels for human disease [13]. Other animal models, often involving mice [14], [15] and guinea pigs [16], [17], are limited by major differences in immune responses with humans [18]. Additionally, they have usually relied on the use of other chlamydial species such as *Chlamydia caviae*, *psittaci*, or *muridarum*, which may be evolutionarily separated from *Ct* and are adapted to their respective hosts.

## Methods

Ethical approval was not required for this review article. References were identified through searches of PubMed for articles published at any date, by use of the terms “trachoma” and “immunology,” “pathogenesis,” “pathology,” “scarring,” or “histology.” Articles resulting from these searches and relevant articles cited in those articles were reviewed. Articles published in English were included. Relevant human studies, which are the focus of this review, are summarised in Tables S1, S2, S3, S4, S5, S6 in Supporting Information S1.

## Clinical Features, Natural History, and Infection

**Figure 1:**
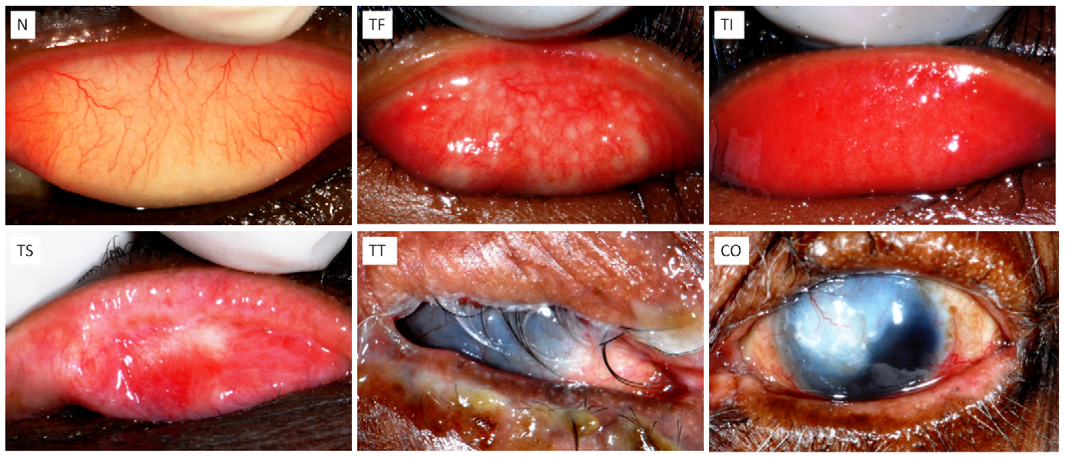
Clinical features and grades of trachoma. N, normal; TF, Trachomatous inflammation–follicular; TI, Trachomatous inflammation–intense; TS, trachomatous scarring; TT, trachomatous trichiasis; CO, corneal opacity.

Infection with *Ct* causes chronic inflammation of the conjunctiva (conjunctivitis) or “active trachoma,” which is characterised by follicles (subepithelial collections of lymphoid cells appearing as small, yellow-white elevations) and papillae (engorgement of small vessels with inflammatory conjunctival thickening). Infection may be detected in the absence of clinical disease, and conversely, active disease may be present without detectable current *Ct* infection [19]. While this may appear paradoxical, there are a number of reasons to explain these findings, including how sensitive the diagnostic test is compared to the clinical examination, and also the kinetics of the disease [19], [20]. These include a latent phase with infection before the onset of clinical signs and a recovery phase where infection has been cleared but clinical signs persist. Children experiencing repeated episodes of infection are at increased risk of subsequent conjunctival scarring, which causes in-turning of the eyelids (entropion) so that the eyelashes scratch the cornea (trichiasis). Eventually sight is lost as irreversible corneal opacification develops from a combination of insults, which compromise the ocular surface (trichiasis, dryness, secondary bacterial, or fungal infections). Clinical features are shown in Figure 1. The scarring complications of trachoma usually develop slowly over many years. Several longitudinal studies support this pathway to blindness, although there is considerable variation in reported disease progression rates, possibly reflecting differences in both the natural history in different populations and in study methodology (Table 1).

**Table 1:** Summary of the incidence and progression rates of trachomatous scarring.

Progression Factor	Sample Size	Follow-Up Interval	Rate	Setting	Prospective Design?	Associated Factors
Incident conjunctival scarring [28], [151]	367	5 y	20.4%	Tanzania	Yes	In children <10 y: active disease/persistent infection; female gender; age
Incident conjunctival scarring [24]	236 (age <7 y)	7 y	29.2% versus 9.6%	Tanzania	No	Higher rate was in children with severe-constant active disease; female gender; age
Worsening of conjunctival scarring [151]	85	5 y	47.1%	Tanzania	Yes	Not specified
Worsening of conjunctival scarring [25]	213^a^	14 y	68.5%	Tunisia	No	Active disease; household density
From conjunctival scarring to trichiasis [26]	523 (all women)	7 y	9.2%	Tanzania	No	Active disease; chlamydial infection; increasing age
From conjunctival scarring to trichiasis [152]	297	12 y	6.4%	Gambia	No	Mandinka ethnicity
From conjunctival scarring to trichiasis [27]	4,898	5 y	3.2%–15.1%	Tanzania	No^b^	Increasing age
From minor to major trichiasis [153]	55	1 y	33%	Gambia	No	None mentioned
From minor to major trichiasis [27]	75	4 y	37%	Gambia	No	Conjunctival inflammation
From unilateral to bilateral trichiasis [153]	46	1 y	46%	Gambia	No	Baseline pannus; hot ash as an aid to epilation
From conjunctival scarring +/− trichiasis to corneal scarring [152]	302	12 y	6.0%	Gambia	No	Baseline trichiasis
From trichiasis to corneal scarring [27]	211	4 y	7.6%	Gambia	No	Increasing trichiasis severity; conjunctival inflammation
From trichiasis to corneal opacity [154]	4,898	10 y	27.2%–53.5%	Tanzania	No^b^	Increasing age
Worsening of corneal scarring [153]	96	1 y	34%	Gambia	No	Conjunctival inflammation; bacterial growth

a Including 82 people with no scarring at baseline.

b Estimated incidence rates based on age-specific prevalence of scarring, trichiasis, and corneal opacity among women.

A key question is why only a minority of people living in trachoma endemic regions develop severe scarring complications. It is likely that the answer to this is a complex interaction between the individual's lifetime burden of infection and their local immune response. Risk factors for trachoma have been extensively reviewed elsewhere [19], [21]–[23] and include poor facial hygiene with unwashed ocular/nasal secretions, crowded living conditions, flies (which act as a vector for the disease), and migration between communities. Severe, persistent, or recurrent conjunctival inflammation is strongly associated with the risk of scarring complications [24]–[28]. Females are at increased risk of conjunctival scarring and trichiasis, possibly from greater lifetime *Ct* exposure through close contact with children [21], [29]. In regions where trachoma is highly endemic, scarring sequelae are more common and occur at a younger age [30], [31]. There is good evidence that the immune response to *Ct* is partly determined by host genetic variations, with several studies from different populations having identified genetic polymorphisms related to immune system function that are associated with trachomatous scarring. These include polymorphisms in interferon-γ (IFNγ), tumour necrosis factor-α, and matrix metalloproteinase (MMP) 9 (Table S1) and indicate the importance of host immunogenetic variations in modifying the risk of developing blinding disease.

Trachoma was initially thought to result from a single episode of infection [32]. However, the importance of repeated re-infection in the pathogenesis of scarring disease is now supported by various lines of investigation: animal models require repeated inoculation to induce disease [9], [10], absence of scarring following a single inoculation in human volunteer studies [32], and cohort studies from trachoma-endemic areas demonstrating repeated infection [20], [28], [33]. However, in vitro studies have shown that persistent, nonreplicating forms of *Ct* develop in response to various stress stimuli (penicillin, IFNγ, and iron or nutrient depletion) [34]. The relevance of these findings to human infection is not known. There is very limited in vivo evidence for persistent ocular infection [35]. An important observation about *Ct* infection and ocular disease is that infection is infrequently detected in older adults, who are the group in which progressive scarring and blinding complications usually develop [27], [36]–[38]. This is partly explained by the shorter duration of infection with increasing age [39]. However, other factors may also be important in scarred conjunctiva, such as nonchlamydial bacterial infection (discussed below).

## Histopathology

Histological examination of conjunctival biopsy samples from subjects with trachoma has given some insights into the disease process (Table S2). However, interpretation of studies conducted to date is limited by the relatively small sample sizes involved and few or no control participants. Comparison with nontrachomatous subjects is essential to establish background conjunctival morphology and cellular compositions in these trachoma-endemic areas, as this may differ markedly from those populations where trachoma is not endemic.

### Active Disease

**Figure 2:**
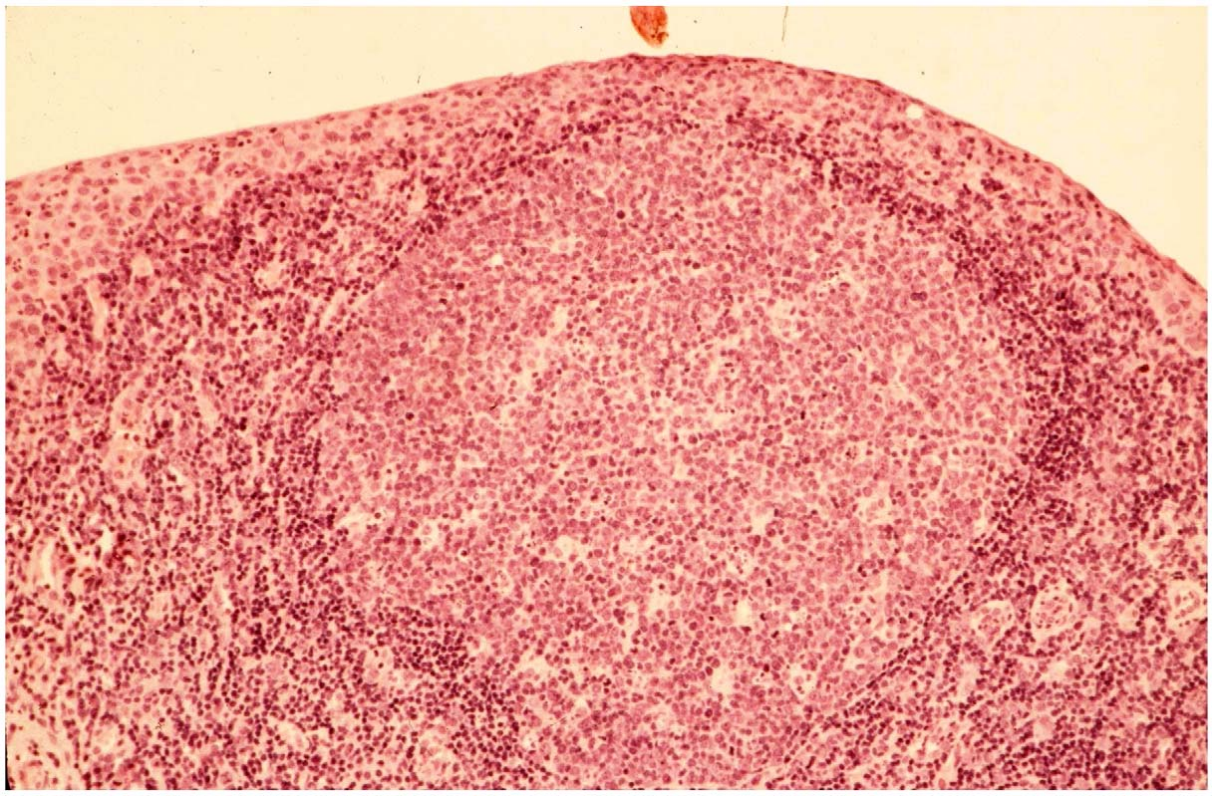
Histological section of the conjunctiva from a child with active trachoma. A subepithelial follicle is seen. Kindly provided by Professor A. El-Asrar, King Saud University.

**Figure 3:**
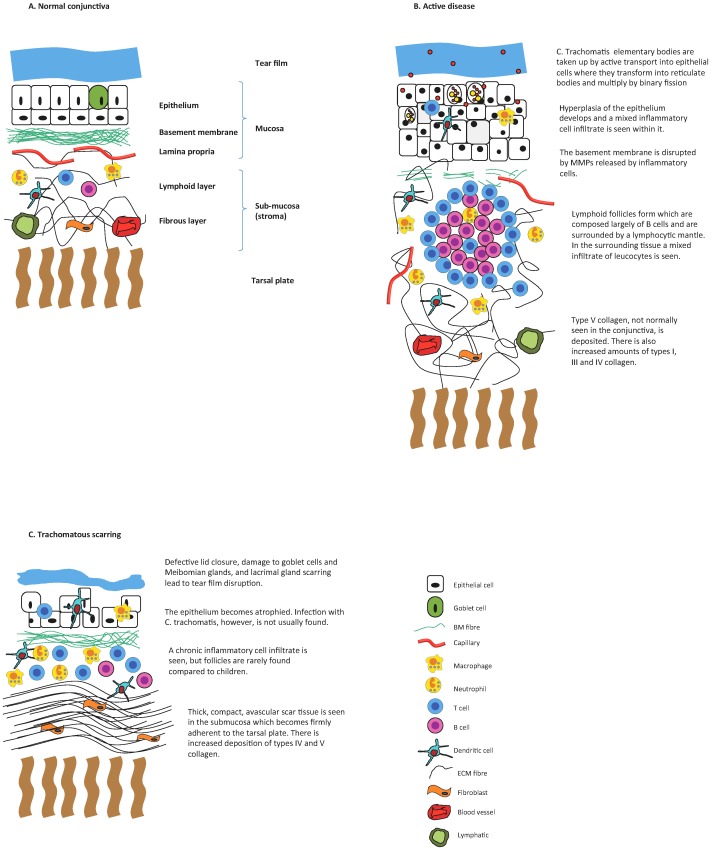
Schemas of normal conjunctiva, active trachomatous disease, and trachomatous scarring.

Histological studies of conjunctival biopsies from children with active trachoma found mild to moderate epithelial hyperplasia with a mixed inflammatory infiltrate consisting of many macrophages with some T cells and polymorphonuclear leucocytes (Table S2) [40]–[42]. Dendritic cells are seen in the deeper epithelium and underlying stroma. Plasma cells form a band directly beneath the epithelium and around accessory lacrimal glands. Lymphoid follicles, the clinical and pathological hallmark of active trachoma, are found in the stroma (Figure 2). In children these consist mainly of B cells with some macrophages and T cells and are surrounded with a lymphocytic mantle. Around the follicle, a diffuse infiltrate consisting of a mixed population of leucocytes (T cells, neutrophils, macrophages, mast cells, and eosinophils) is seen. Schematic diagrams of normal conjunctiva and active disease are shown in Figure 3.

### Scarring Disease

**Figure 4:**
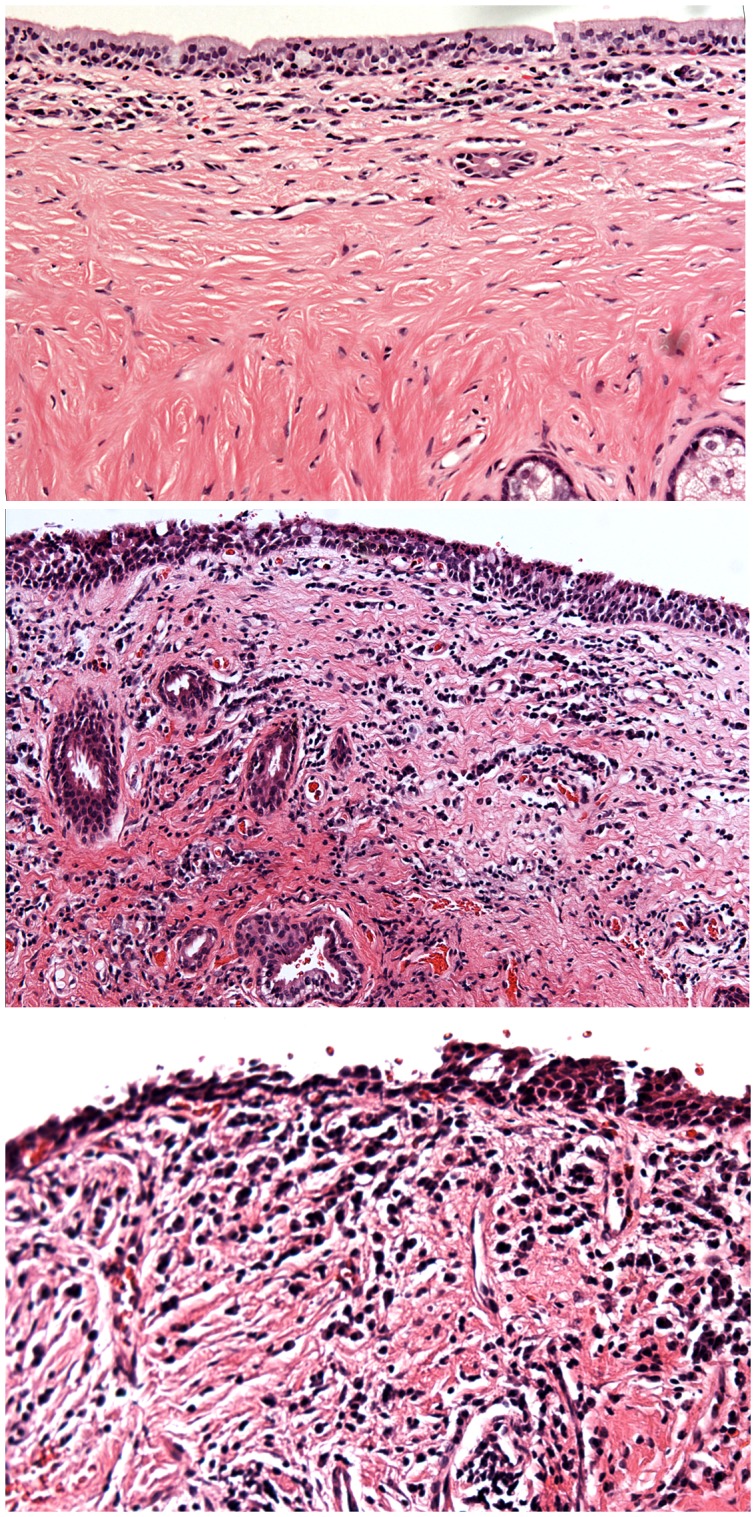
Histological sections of healthy conjunctiva and trachomatous scarring. (Top) Healthy. (Middle and bottom) Scarred, note disruption of the epithelial and connective tissue morphology, and an increased inflammatory cells.

Adults with conjunctival scarring have an epithelium that may show either squamous metaplasia or atrophy with focally denuded areas replaced by fibrinous exudate and cellular debris (Figure 4) [42], [43]. A chronic inflammatory cell infiltrate is found, most marked in the substantia propria with lymphocytes predominating [40], [44]. CD4+ and CD8+ T cells are present, and these generally outnumber B cells. Follicles may be seen histologically in very inflamed subjects, even when absent clinically; these contain monocytes, macrophages, and plasma cells but lack the germinal centres or centrally located B cells seen in follicles of children. Underneath the epithelium, the stroma is replaced by abnormal, compact, thick, and mostly avascular scar tissue [43]. This subepithelial fibrous membrane has fibres parallel to the surface epithelium and is firmly adherent to the tarsal plate (Figure 3).

## Resolution of *Ct* Infection

A number of immune-mediated responses have been demonstrated to *Ct* infection that are thought to be important in clearing infection. The area requires further clarification, however, with many of these responses yet to be unequivocally associated with resolution of infection in human *Ct* infection.

### Epithelial Cells Mount a Pro-Inflammatory and Chemotactic Response

Several in vitro studies of *Ct*-infected epithelial cell lines have found that this infection provokes pro-inflammatory responses with epithelial cells producing inflammatory and chemotactic cytokines such as interleukin-6 (IL-6), IL-8, growth-regulated oncogene α (GROα), and granulocyte macrophage colony-stimulating factor (GM-CSF) [45]–[47]. The secretion of these factors was prolonged and required synthesis of chlamydial proteins. In contrast, other bacterial species provoked a rapid but transient cytokine induction [45].

Human studies are consistent with the epithelium-producing pro-inflammatory factors constituting an initial innate response to *Ct*. Immunohistochemistry of conjunctival epithelium from children with active trachoma has shown IL-1 expression [48]. Gene expression studies on conjunctival surface swab samples find enriched expression of multiple pro-inflammatory/chemotactic factors (Table S3). In active trachoma, the epithelium shows increased Major Histocompatibility Complex (MHC) class I expression (found at a lower level in normal conjunctiva) and induction of MHC class II (not normally found at this site) [41], [49]. There is also evidence for natural killer (NK) cell and neutrophil recruitment and activation in active disease/infection as part of an acute inflammatory response [16], [50], [51].

### Importance of a CMI Response and IFNγ Production

The results of animal studies of genital chlamydial infection suggest that resolution of infection is dependent on a Type-1 CD4+ T-helper lymphocyte (Th1) response-mediated primarily through IFNγ. Congenitally athymic (nude) mice are unable to clear *C. muridarum* genital tract infection [52]. However, adoptive transfer to nude mice of CD4+ or CD8+ T cells derived from normal mice previously infected with *C. muridarum* resolved the infection [53]. The CD4+-enriched cell line was much more efficient at clearing infection. However, recent murine studies using *Ct* instead of *C. muridarum* as the infectious agent did not find that the absence of CD4+ cells altered the course of infection and limitations of animal models of *Ct* infection need to be acknowledged [54]. Although CD8+ T cells can help resolve infection, they seem less important in animal models, as they were not an absolute requirement for clearing infection [55].

IFNγ is produced by several cell types including Th1 lymphocytes, CD8+ cytotoxic T lymphocytes (CTLs), and NK cells. The production of IFNγ characterises the Th1 response and has several antichlamydial actions, which probably play a central role in clearing infection. IFNγ induces indoleamine-2,3-dioxygenase (IDO) expression, which lowers the intracellular pool of the essential amino acid tryptophan, which is necessary for chlamydial metabolism [56], [57]. IFNγ can also up-regulate inducible nitric oxide synthase (iNOS), which may help control *Ct* and protect against chronic sequelae in murine models [58]. In vitro, chlamydial growth can also be limited by IFNγ-mediated intracellular iron depletion [59].

Several human studies point to the importance of the CMI response to ocular *Ct* infection, although direct longitudinal evidence that resolution is IFNγ-dependent is limited. Biopsies from children with active trachoma show heavy cellular infiltrates with both CD4+ and CD8+ lymphocytes (Table S2) [41]. Children who resolved active disease (associated with infection) had stronger peripheral blood lymphoproliferative responses to *Ct* antigens compared to children with persistent disease (Table S4) [60]. Gene expression studies have shown increased expression of *IFNγ*, *IL12p40*, *Perforin*, *IDO*, *IL-4*, *IL-10*, and forkhead box p3 (*FOXP3*) in infection and/or disease, with levels generally being highest if both were present (Table S3) [33], [50], [61], [62]. Increased expression of *IFNγ*, *IL-10*, and *FOXP3* has been associated with longer episodes of infection [63]. A recent study using *Ct* elementary bodies (EBs) to stimulate PBMC from individuals exposed to ocular infection showed that cells with a classical NK cell phenotype (CD3-CD56+) were an additional major source of IFNγ [51].

## Protective Immunity to *Ct*

Human volunteer studies of ocular *Ct* infection have provided some evidence that a partial, protective immune response develops [64]–[66]. Conjunctival inoculation with *Ct* led to a characteristic follicular/papillary conjunctivitis in nearly all volunteers after 2–14 d. However, when previously infected individuals were rechallenged with the same serovar, there was an attenuated clinical response with reduced re-isolation rates. This apparent immunity to re-infection was serovar-specific: if a different serovar was used, then disease and infection levels were similar to the primary infection. Presumed complete immunity after primary infection, in which no clinical signs developed and the organism was never detected, despite multiple rechallenge inoculations, was rare. Despite the concerns over the induction of presumed delayed-type hypersensitivity (DTH), the trachoma vaccine trials in the 1960s did demonstrate short-term, serovar-specific immunity could be induced [67], [68]. Trachoma models in nonhuman primates also showed that, after recovery from a primary infection, on secondary challenge there was less severe disease that resolved more rapidly [9]. With regular weekly re-inoculations, it also became progressively more difficult to detect the organism, suggesting the development of protective immunity. The human studies discussed above were conducted over 40 years ago and need to be interpreted in that context. Ethical considerations, however, limit contemporary studies to being of an observational design.

A longitudinal study in which a cohort from a trachoma endemic population was regularly assessed for disease and infection found the duration of both shortened considerably with increasing age [39], [69]. This is thought to be the result of an increasingly effective protective immune response developing with repeated exposure to *Ct*, although other factors could also have been important, such as behavioural changes. Another study has shown that in 4- to 15-y-olds, there was evidence of age-related modulation of cytokine expression, with older individuals tending to have greater expression of IFNγ, IDO, and *IL-10* [62]. The clinical manifestations also appeared to be modulated: adults experienced short bursts of intense disease and children more prolonged episodes of follicular disease [39].

Acquired T-cell–dependent CMI responses, as outlined above, probably contribute to protection against or more rapid resolution of re-infection, although direct evidence for this from human trachoma studies is limited. Fading immunity in animal models is correlated with a reduction in CD4+ T cells, which leave the genital tract following resolution of infection [70]. A study of the incidence of genital tract infection amongst sex workers in Nairobi, Kenya, found that IFNγ production by PBMC stimulated with cHsp60 was associated with reduced risk of re-infection compared to women without such responses [71].

The importance of humoral immunity to *Ct* is unclear. Several human studies have detected antichlamydial antibodies in tear fluid and serum (Table S5). However, in a longitudinal study, antichlamydial IgG in tears from clinically normal individuals was associated with an increased incidence of subsequently developing clinically active trachoma [72]. In contrast, there was an opposite (nonsignificant) trend with antichlamydial IgA. Individuals with conjunctival scarring have been found to have significantly higher plasma titres of antichlamydial IgG and lower titres of IgA compared with normal controls [73].

However, a consistent picture on the role of antibody in protection from trachoma has not emerged from these studies. What is clear is that the overall level of the antibody response can be a useful proxy of the lifetime exposure to ocular infection. Within the overall antibody response, a profile of specific antibodies directed against a limited number of individual antigens are associated with the presence or absence of clinical signs of disease [74]. However, how such antibodies mediate protection and pathology requires elucidation.

Various animal models of genital tract infection have yielded a mixed pattern of results on the relative importance of the humoral response. Some have shown that B-cell–deficient mice can resolve a primary chlamydial infection and are only slightly delayed in clearing secondary infections, compared to control mice [75], [76]. However, other studies found that B-cell–deficient mice that also had their CD4+ cells depleted were completely unable to control secondary infection, whereas mice depleted of CD4+ cells alone only showed slight delay in clearing secondary infection [77], [78]. In addition, transfer of monoclonal antibodies to *Ct* major outer membrane protein (MOMP) and LPS into B-cell–deficient, CD4+ T-cell–depleted mice restored the ability of these mice to control secondary infection [79]. Antibodies to polymorphic membrane protein D (PmpD) have also been shown to be neutralising in vitro [80]. However, whether similar protective effects occur in human ocular infection is unknown.

## Immunopathogenesis of Trachoma

In 1974 Silverstein proposed that tissue damage and scarring sequelae in trachoma are the result of a chronic, immunopathogenic response to an otherwise seemingly innocuous, superficial infection [81]. He observed that *Ct* infection itself is limited to a small minority of epithelial cells and is not particularly cytopathic, with cells accommodating large chlamydial inclusion bodies. The infection can be quite prolonged, suggesting that the immune response is relatively inefficient or that *Ct* is effective at avoiding immune responses. At the tissue level, the inflammatory infiltrate and subsequent scarring is in the conjunctival stroma, below the level of the epithelium, where the infection is located.

A key clinical observation pointing to the immunopathogenic nature of trachoma is that visible conjunctival inflammation is frequently found in the absence of detectable *Ct* [19], [20], [33], [61], [82]. In longitudinal studies, discrete episodes of active disease persist long after the associated initiating infectious episode has resolved [39]. In one trachoma vaccine trial, the incidence of trachoma was higher in the vaccinated group and another reported a possible increase in disease severity [68], [83].

Monkey models of *Ct* infection support the importance of immunopathology in trachoma [9]. Treatment of infected animals with corticosteroids markedly reduced the inflammation, and in monkey vaccine studies, rechallenge was associated with more severe inflammatory disease and scarring complications [84], [85].

Several theories for the pathogenesis and scarring caused by chlamydial infection have been proposed, none of which entirely explain the observed data. Stephens helpfully subdivided these into two broad paradigms: the “cellular paradigm” in which the response of the epithelial cell layer is central and sufficient to explain the pathology, and the “immunological paradigm” in which cell-mediated immunity causing either DTH or autoimmune mimicry explains disease pathology [86]. These processes are not mutually exclusive and may both contribute.

## The “Immunological Paradigm” and Trachoma

The “immunological paradigm” suggests that tissue damage and fibrosis in chlamydia-related disease results from CMI responses against specific chlamydial antigens [46], [55], [81], [86]. It argues that specific T-cell responses, which are important in clearing *Ct* infection, also cause collateral tissue damage. There are two major lines of investigation that have explored this hypothesis, which are discussed below.

### DTH and cHsp60

During early trachoma vaccine studies on nonhuman primates, it was observed that the disease phenotype could be more severe on rechallenge. This led the investigators to propose that a DTH reaction to chlamydial antigens was responsible for the disease [10]. Subsequently, in a guinea pig model of ocular infection, a *Ct* antigen, extracted using the detergent triton X-100, was able to provoke an inflammatory reaction in animals previously infected with *Ct* [87]. The active component was identified as the 57 kDa protein, cHsp60 [88], [89]. Similar experiments were conducted in cynomolgus monkeys, with comparable results [11]. There was some concern that the conjunctival inflammatory response observed in these monkeys might have been partly provoked by the triton X-100 detergent itself, as repeated installation of the buffer alone also led to significant inflammation. However, when experiments were repeated with recombinant cHsp60, inflammation was provoked in guinea pig eyes and in the subcutaneous abdominal salpingitis pocket model in monkeys [88]–[91]. Interpretation of the role of cHsp60 from these animal models is difficult, since a further study showed that initial immunisation with recombinant antigen in guinea pigs did not produce exacerbated disease after ocular infection with viable organisms but rather may have led to some protective effect [92].

Human studies on cHsp60 have delivered mixed results on its role in pathogenesis. Antibodies to cHsp60 are associated with trachomatous scarring, pelvic inflammatory disease, and tubal infertility [93]–[95]. It is unclear, however, whether these antibodies have a pathogenic role or are simply markers of previous infection. Other studies have suggested that immune responses to cHsp60 may even be protective: examination of PBMC proliferation responses to chlamydial antigens in trachoma endemic populations found that conjunctival scarring was associated with weaker responses to cHsp60, while the resolution of infection was associated with increased responses [60], [73]. Also, as noted above, IFNγ production by PBMC stimulated with cHsp60 is associated with protection against subsequent genital tract re-infection [71]. Overall, there is little evidence from human studies to suggest that scarring trachoma is the result of a DTH reaction to cHsp60.

### Differential Activity of CD4+ T-Cell Subsets

A Th2-dominated response has been linked to the development of scarring complications in some infectious diseases such as schistosomiasis [96]. However, there is little evidence for a similar role in trachoma. Conjunctival gene expression profiling in children with active disease and *Ct* infection found an increase in both Th1 (*IFNγ*) and Th2 (*IL-4*) cytokine expression (Table S3) [33], [61]. Adults with scarring, compared to controls, have reduced lymphoproliferative responses and IFNγ production in response to stimulation with *Ct* EB and some antigens, but an increased number of IL-4–producing cells in response to cHsp60 [73], [97]. This seems to support the hypothesis that individuals with scarring may have weaker Th1 cell-mediated responses to *Ct*, leading to prolonged infection and inflammation possibly as a result of Th2 responses with pro-fibrotic effects. However, recent studies comparing the conjunctival transcriptome by microarray, and RT-PCR expression of selected targets, in subjects with scarring trachoma and matched controls found no evidence for Th2 responses and only indirect evidence of increased expression of genes associated with Th1 cells [98]–[100]. Furthermore, the expression of *IL-13* was lower in individuals with established conjunctival scarring and inflammation, compared to healthy controls. Additionally, Th2 cytokine levels in tear fluid were not increased in scarred individuals (Table S6) [101].

The role other types of CD4+ T cells play in trachoma pathogenesis has received little attention. One study showed that the expression of *FOXP3*, used as an indicator of regulatory T cells, was increased in children who had clinical signs of trachoma but in whom infection had resolved [62]. However, whether this role is beneficial, by dampening the immune response and subsequent tissue damage once infection has been cleared, or detrimental, by impeding removal of infectious agent, is unknown. The expression of *IL-17A*, suggestive of Th17 cell activity, has been found to be significantly increased in active trachoma (discussed below) [50], [102], [103].

## The “Cellular Paradigm” and Trachoma

In the “cellular paradigm,” tissue damage and scarring are thought to be driven by infected epithelial cells, which serve as early innate system responders [46], [86]. Pro-inflammatory chemokines, cytokines, and growth factors produced by *Ct*-infected epithelial cells initiate the recruitment of inflammatory immune cells (neutrophils, macrophages, and NK cells). Infected epithelial cells express multiple factors, which also promote the CMI response and are chemotactic for lymphocytes. The “cellular paradigm” does not differentiate between damage resulting from professional innate immune cells, such as neutrophils and monocytes, and adaptive immune cells [46]. However, it does propose that chronic inflammatory responses, tissue damage, and fibrosis in *Ct*-related disease is largely driven from the epithelium rather than by CMI. There is now increasing evidence that innate immune responses are a prominent feature of responses in trachoma and likely interact with the adaptive response.

Animal studies have shown an influx of neutrophils into genital tract tissue following *Ct* inoculation [104]. Neutrophils were not required to clear infection, however the intensity of the neutrophil infiltrate was related to subsequent fibrotic sequelae [105]. A guinea pig model of trachoma examining neutrophil depletion showed no effect on the burden of infection, however there was less clinical inflammation and fewer mucosal erosions histologically [16]. In this study, adaptive immune responses were also affected with reduced CD4+ and CD8+ cell recruitment and changes in the expression of various cytokines and chemokines such as decreased *CCL5*. This study only evaluated animals for up to 7 days, and once again caution needs to be exercised in extrapolating results of animal models to humans. However, it does provide an insight into the role of innate immunity in trachoma pathogenesis and how this might partly be mediated through its interaction with adaptive responses.

The production of pro-inflammatory cytokines in response to *Ct* infection is mediated in part through recognition of pathogen-associated molecular patterns (PAMPs) by Toll-like receptors (TLRs) and other pattern recognition receptors (PRRs) [106]. While TLR2 knockout (KO) mice were able to eradicate infection in a similar manner to control mice, they had reduced TNFα and CXCL2 [107]. Of particular note is the observation that TLR2-KO mice also had a marked reduction in late oviduct pathology. Human genetic studies on TLR polymorphisms have yet to identify significant associations with chlamydial diseases [108]–[110].

In children with active trachoma, IL-1 has been found in the surface epithelial cells of cases, but not controls [48]. This might promote recruitment of innate immune cells, including neutrophils and macrophages, which are indeed seen in large numbers in the subepithelial substantia propria of children with active trachoma [40], [41]. In vivo confocal microscopy of the conjunctival surface has shown that most of the cellular infiltrate in active and scarring trachoma is concentrated just below the epithelium, supporting the importance of the epithelial cell layer as a source of chemotactic factors [111].

For both active and scarring trachoma conjunctival transcriptome, studies found prominent innate immune responses [50], [99]. In children with active disease and/or *Ct* infection, there was marked enrichment of neutrophil and NK-cell-related transcripts [50]. In addition, several PRR and chemokines including the neutrophil chemotactic factor *CXCL5* were increased [103]. In adults with scarring and little *Ct* infection, there was also strong evidence for an innate immune response, with some of the most abundant increases in gene expression found for pro-inflammatory mediators such as Psoriasin-1 (*S100A7*), *IL-1B*, and *CXCL5* [99], [100]. These factors induce neutrophil chemotaxis and were particularly increased in inflamed cases. The importance of the chemokine response in trachoma is further supported by the finding that genetic variation across the *IL-8* locus, defined by haplotypes of multiple single nucleotide polymorphisms (SNPs), was associated with scarring [112].

TNFα, while not specific to innate immune processes, is a key cytokine in acute inflammation and has been associated with scarring trachoma in several studies: a SNP in the *TNFA* promoter region, elevated levels in tear fluid, and increased secretion from peripheral blood mononuclear cells (PBMCs) from scarred subjects stimulated with EBs [101], [113]–[115]. Increased transcript levels of *TNFA*, as well as *IL-1B*, have also been associated with active disease/infection [33], [61], [116].

There is increasing evidence that nonchlamydial bacterial infection could play a role in the pathogenesis of trachoma and that this is likely to be through innate mechanisms. While infection with *Ct* is often found in children with active trachoma, it is only rarely identified in adults, as discussed above. Infection with bacteria other than *Ct*, however, is more common in individuals with conjunctival scarring, trichiasis, or its recurrence after surgery, compared to controls, and it is also more common in active disease [36], [37], [117]–[119]. This nonchlamydial bacterial infection is associated with elevated expression of a number of antimicrobial peptides, pro-inflammatory mediators, and modifiers of the extracellular matrix (ECM) including *CXCL5*, *S100A7*, *DEFB4A*, *IL-1B*, and *MMPs 1*, *9*, *10*, and *12* [100], [103], [120]. It is tempting to postulate that the conjunctiva of individuals who have been repeatedly infected with *Ct* undergoes morphological/immunological changes, rendering it susceptible to nonchlamydial bacterial infection, and that this subsequent infection causes an exaggerated inflammatory response with propagation of the scarring process. However, it is also possible that nonchlamydial bacterial infection may itself promote *Ct* infection or the different types of infection may act synergistically.

## A Role for IL-17A in Trachoma?

Neither the “immunological” nor “cellular paradigms” completely accommodate the published data regarding trachoma pathogenesis. Initial studies suggest that IL-17A may be important in trachoma pathogenesis [50], [103]. IL-17A is the signature cytokine of Th17 cells, a CD4+ T-cell population that act in an antigen-specific manner [121]. However, it is also produced by several other cell types, notably innate immune cells (γδ T cells, NK cells, macrophages, neutrophils), and it can contribute to innate inflammatory responses [122], [123]. The IL-17A receptor is found on various cell types including dendritic cells, lymphocytes, epithelial cells, and fibroblasts [124]. IL-17A is pro-inflammatory and plays an important role in host immunity to extracellular and some intracellular pathogens [125]. Recently, it has become apparent that IL-17A may contribute to fibrosis through several mechanisms including epithelial-mesenchymal transition (EMT) and increased collagen production in a TGFβ1-dependant manner [126]. Infection of mice with *Mycobacterium tuberculosis* followed by repeated BCG injections led to an IL-17A–dominated response, which was refractory to regulation by IFNγ [127]. This caused extensive lung tissue damage by neutrophils that could be reduced by anti-IL17A antibody. These observations from TB may have parallels with trachoma, with IL-17A playing a major part in immunopathology, and could have implications for vaccine strategies.

## Tissue Damage and Fibrogenesis in Trachoma

Scarring develops when normal tissue architecture is disrupted and replaced by excessive connective tissue through the abnormal accumulation of ECM components. Tissue damage can be mediated through a variety of cell types and mechanisms. Neutrophil infiltration appears important in trachoma: they have been identified in conjunctival biopsies of trachomatous tissue, they produce toxic reactive oxygen and nitrogen species that damage host tissue in animal models of genital tract infection, and they can also produce MMPs [48], [128]. Macrophages are also found in trachomatous tissue and may be important effector cells for tissue damage in chlamydial infection [48].

The matrix metalloproteinases are a group of more than 25 endopeptidases with multiple, complex functions. While MMPs are required for normal tissue homeostasis, there is also evidence that they play a role in the pathogenesis of a range of inflammatory-fibrotic diseases [129]–[131]. An important step is the disruption of the basement membrane, aiding the recruitment of inflammatory cells [132]. MMPs also have wide-ranging effects on inflammatory and immune processes such as modulating chemokine activity and activation of TGFβ, IL-1β, and TNFα. [133]. They are known to be important in a number of ocular surface diseases, and inhibition of MMP activity has been shown to reduce conjunctival scarring after glaucoma surgery [134], [135].

MMP9 in particular has been studied extensively in trachoma. It is part of the neutrophil lysosome and mediates epithelial dissolution associated with infection through degradation of type IV collagen [128]. Mouse models of genital tract infection have shown a reduced rate of scarring sequelae in *MMP9* KO animals and that infection is associated with increased MMP9 in genital tract tissue (determined by zymography and gene expression) [15], [136]. Children with active trachoma have increased amounts of MMP9 (determined by immunohistochemistry, zymography, and gene expression analysis) [61], [137]. Scarring trachoma in adults is associated with increased expression of *MMP9* and a coding SNP that is adjacent to the active binding site of the MMP9 enzyme [61], [100], [138]. Scarring trachoma is also associated with differential expression of *MMPs* 7, 9, 10, and 12 and tissue inhibitor of MMP (TIMP)-1, and recurrence of trichiasis after surgery is associated with an altered *MMP1/TIMP1* transcript ratio [98]–[100], [120].

The production of scar tissue has not been extensively studied in trachoma. However, it probably originates from activated fibroblasts that are stimulated to produce collagen by various pro-fibrogenic mediators (TGF-β, PDGF, CTGF, and bFGF) [48], [103], [139]. Chemokines have also been shown to act as fibrogenic mediators, in particular the CC- and CXC-chemokine families, and various members of these families have been associated with scarring including the pro-fibrogenic CCL18 [96], [99], [101]–[103], [132].

## Programmatic Implications

Trachoma is still endemic in over 50 countries and current trachoma control strategies face major obstacles. With regards to mass antibiotic distribution, the coverage levels in practice are often disappointing; there are concerns that mass distribution of azithromycin may lead to an increase in antibiotic resistance, and the “arrested immunity hypothesis,” put forward in the context of genital tract infection, suggests that shortening the duration of chlamydial infection with treatment results in population-wide reductions in protective immunity [140]–[142]. As well as being potentially the most effective way of reducing blindness from trachoma, a chlamydial vaccine could be very cost effective and have major benefits for genital tract disease caused by *Ct* [143], [144].

The human trachoma vaccine studies in the 1960s using inactivated whole EB tended to show only partial, short-term, serovar-specific protection, which was little better than natural immunity and may have resulted in earlier disease onset in some vaccinated individuals [67], [68], [83], [145]–[148]. Understanding the immunobiology of trachoma, including both how infection is successfully cleared and its pathogenesis, is important in the rational design of an effective vaccine that avoids immunopathology. A recent study employed a plasmid-deficient strain of *Ct* to immunise cynomolgus macaque monkeys [149]. Attenuated organisms were inoculated directly onto the conjunctival surface and caused minimal/absent ocular inflammation, despite repeated innoculation. On rechallenge with plasmid-bearing organisms, previously infected animals shed markedly less infectious organisms than controls, and three out of six animals showed minimal inflammatory changes compared to marked inflammation in all six control animals. These encouraging results, as well as implicating the *Ct* plasmid as an important virulence factor, support further work into vaccine development. While plasmid-deficient strains of *Ct* are naturally isolated from populations at risk of trachoma, the manipulation of virulence factors and the development of vaccine candidates may be helped by recent advances in *Ct* genetic transformation [150].

## Conclusion

The immunology and pathogenesis of trachoma is a challenging but fascinating area of study. Despite considerable efforts, the mechanisms of protective immunity in trachoma remain elusive, although data from animal models suggest these involve IFNγ-dependent CMI responses. Recent data point to the importance of the human innate immune response, epithelial cell responses, EMT, and possible IL-17A involvement in both active disease and the pathogenesis of scarring. The MMPs are important effector enzymes in this tissue damage. There is still a pressing need for further research to better understand this ancient disease. The development of a vaccine would help to overcome the many obstacles that lie in the way of eliminating blinding trachoma.

**Key Learning Points**

- The immune response to *Ct* provides only partial protection against re-infection and is important in causing tissue scarring and blindness.
- Recent evidence has supported the “cellular paradigm” of disease pathogenesis, which states that infected epithelial cell responses drive pathology through the release of various mediators.
- A trachoma vaccine would offer significant advantages to trachoma control, but vaccine development is impeded by a limited understanding of disease mechanisms.

**Key Papers in the Field**

- Stephens RS (2003) The cellular paradigm of chlamydial pathogenesis. Trends Microbiol 11: 44–51.
- Darville T, Hiltke TJ Pathogenesis of genital tract disease due to Chlamydia trachomatis. J Infect Dis 201 Suppl 2: S114–S125.
- Burton MJ, Rajak SN, Bauer J, Weiss HA, Tolbert SB, et al. (2011) Conjunctival transcriptome in scarring trachoma. Infect Immun 79: 499–511.
- Kari L, Whitmire WM, Olivares-Zavaleta N, Goheen MM, Taylor LD, et al. (2011) A live-attenuated chlamydial vaccine protects against trachoma in nonhuman primates. J Exp Med 208: 2217–2223.

## Supporting Information

Supporting Information S1 Table S1: Human trachoma genetic studies; Table S2: Histology and immunohistochemistry studies using human tarsal conjunctival biopsies; Table S3: Quantitative gene expression and microarray studies from human tarsal conjunctival swab samples; Table S4: Lymphoproliferative and cytokine studies using human PBMCs; Table S5: Antibody/B-cell responses from human serum, conjunctival, and tear samples; Table S6: Miscellaneous human trachoma studies.
(DOCX)
