# Supplementary material for: Trachoma: Protective and Pathogenic Ocular Immune Responses to Chlamydia trachomatis
Source: PLoS Negl Trop Dis. 2013 Feb 14;7(2):e2020. doi: 10.1371/journal.pntd.0002020 (PMC3573101; doi:10.1371/journal.pntd.0002020)
Supplement: Supporting Information S1 — Table S1: Human trachoma genetic studies; Table S2: Histology and immunohistochemistry studies using human tarsal conjunctival biopsies; Table S3: Quantitative gene expression and microarray studies from human tarsal conjunctival swab samples; Table S4: Lymphoproliferative and cytokine studies using human PBMCs; Table S5: Antibody/B-cell responses from human serum, conjunctival, and tear samples; Table S6: Miscellaneous human trachoma studies. (DOCX) [file pntd.0002020.s001.docx]

**Table S1*.*** Human trachoma genetic studies.

| **Title** | **Study summary** | **Sample size / Participants** | **Location** | **Key findings** | | **Comments** |
| --- | --- | --- | --- | --- | --- | --- |
|  |  |  |  |  | |  |
| HLA class I and II polymorphisms and trachomatous scarring in a *Ct* endemic population[1] | Case-control study of scarred subjects and controls, measuring HLA class I and II polymorphisms using serotyping and PCR. Chlamydial antibody titres were measured using MIF. | 153 cases and 153 controls. | The Gambia | | Scarring was associated with increased HLA-A28, specifically, the allele subtype A*6802. | Uncorrected p-values presented for serological testing of HLA class I antigens. |
| HLA antigens in Omanis with blinding trachoma: markers for disease susceptibility and resistance[2] | Case-control study of subjects with severe scarring and controls, measuring HLA class I and II polymorphisms using serotyping and PCR. | 50 cases (TT and CO) and 100 controls. | Oman | | Scarring was associated with increased HLA-A32, DR2, DR16 and DQ1, and with decreased HLA-A30, DR4, DR7, DR5 and DR53. | Controls were not matched for age or risk of trachoma (included university students and blood donors). Odds ratios would have been more appropriate to present than risk ratios. |
| Scarring trachoma is associated with polymorphism in the TNF-α gene promoter and with elevated TNF-α levels in tear fluid[3] | Case-control study of subjects with scarring and controls analysing SNPs of TNF-α using PCR, and HLA polymorphisms using serotyping. TNF-α level in tear fluid measured. | 153 cases and 153 controls. | The Gambia | | Scarring independently associated with increased TNF-α-308A and HLA-A*6802 (which were themselves also associated). Elevated tear TNF-α was associated with scarring and infection, but not the SNPs. | Same subjects as previous study in The Gambia.[1] |
| Polymorphisms in candidate genes and risk of scarring trachoma in a *Ct* endemic population[4] | Case-control study of cases with scarring and controls, analysing SNPs of IL-4, IL-10, TNF-α and MBP using PCR. | 238 cases and 239 controls. | The Gambia | | In the Mandinka ethnic sub-group scarring was associated with increased IL-10-1082G. | TNF-α-308A not analysed. Overlap of subjects with previous study.[1] |
| Risk of trachomatous scarring and trichiasis in Gambians varies with SNP haplotypes at the INF-γ and IL-10 loci[5] | Case-control study of cases with scarring and controls, analysing SNPs of INF-α and IL-10 using PCR. The potential effect of LD was examined by analysing SNP haplotypes. | 344 cases with TS and 344 controls; 307 cases with TT and 307 controls. | The Gambia | | Scarring was associated with increased IFN-γ+3234C (TS only), IL-10-3575A and IL-10+5009G. There was a stronger association with TT compared to TS cases for the IL-10 alleles. SNP haplotypes were also associated with scarring. | Overlap of subjects with previous study.[4] |
| A coding polymorphism in MMP-9 reduces risk of scarring sequelae of ocular *Ct* infection[6] | Case-control study of cases with scarring and controls, analysing for SNPs and SNP haplotypes of MMP-9 using PCR. | 344 cases with TS and 344 controls; 307 cases with TT and 307 controls. | The Gambia | | Scarring was associated with decreased MMP-9-Q279RG, with a stronger association for TT compared to TS cases. The association was only with the heterozygous genotype. Haplotype analysis supported a direct association with the SNP. | The SNP adjacent to the active site of the enzyme, potentially having a direct effect on function. Although a coding SNP, direct evidence of a functional difference not yet proven. Same subjects as previous study[5] |
| Genetic variation at the TNF locus and the risk of severe sequelae of ocular *Ct* infection in Gambians[7] | Case-control study of cases with scarring and controls, analysing SNPs and SNP haplotypes of TNF and neighbouring I*_K_*BL and LTA alleles using PCR | 344 cases with TS and 344 controls; 307 cases with TT and 307 controls. | The Gambia | | Scarring in TT cases was associated with increased TNF-α-308A, I*_K_*BL-63T and LTA+252G. The TNF-α association was only with the heterozygous genotype. | Same subjects as previous study.[5] A weaker association for TNF-α-308A was found than in the earlier study despite a larger sample size. |
| Susceptibility to sequelae of human ocular chlamydial infection associated with allelic variation in IL10 cis-regulation[8] | Relative allelic expression of IL-10 transcript levels were measured in individuals with active disease who were heterozygous for an IL-10 haplotype previously shown to be associated with scarring. | 23 children with active disease | The Gambia | | The haplotype associated with scarring generated relatively more IL-10 transcripts. |  |
| Identification of novel SNPs in inflammatory genes as risk factors associated with TT[9] | Case-control study analysing for SNPs in IL-1α, IL-1β, IL-4, IL-4R, IL-5Rα, IL-6, IL-9, IL-10, IL-13, ADRB-2, C3, C5, CCR-2, CCR-3, CCR-5, CD14, CSF-2, CTLA-4, ICAM-1, FCER-1β, LTA, LTC-4S, NOS-2A, NOS-3, SDF-1, SELE, SELP, SCYA-11, TCF-7, TGF-1β, TNF-α, UGB, VCAM-1 and VDR. | 135 cases with TT and 232 controls. | Nepal | | After adjusting for age, sex, inflammation and *Ct* infection: scarring in TT cases was associated with increased IL-9+T13MC, TNF-α-308G, LTA-252G and VCAM1-1594T (most of which were for heterozygous genotypes). | Unclear subject selection with mean age of active cases being 35 years. The results for active cases and TS cases are not shown or discussed. The p-value was not adjusted for multiple testing. |
| HLA-B, DRB1, and DQB1 allotypes associated with disease and protection of trachoma endemic villagers[10] | Case-control study measuring HLA-DRB1, DQB1 and B polymorphisms in cases and controls using PCR. | 21 older girls and women with TT (+/- TI) and 77 controls; 11 children with persistent infection and 11 controls. | Tanzania | | Scarring was associated with increased HLA-B*7 and HLA-B*8 and with decreased DR-B*11. | 12 of the “controls” in the TT study had TF. Buccal swabs used for DNA collection so that low resolution HLA typing only was possible. The p-value was not adjusted for multiple testing. |
| Innate immunity in ocular *Ct* infection: contribution of IL8 and CSF2 gene variants to risk of trachomatous scarring in Gambians[11] | Case-control study using LD to measure risk associations across chromosomal regions including the IL-8 and CSF-2 genes. | 344 cases with TS and 344 controls; 307 cases with TT and 307 controls. | The Gambia | | Scarring was associated with variation at the IL-8 and CSF-2 loci. | TS and TT subjects divided between different studies. The p-value was not adjusted for multiple testing. |
| Host genetic contribution to the cellular immune response to Ct: heritability estimate from a Gambian twin study[12] | Twin study measuring lymphoproliferative responses to Ct EBs | 19 monozygotic and 45 dizygotic twin pairs. | The Gambia | | Genetic factors were estimated to contribute to 39% of the variation in responses. | The p-value of the heritability estimate was of borderline significance at 0.07. |

**Table S2*.*** Histology and immunohistochemistry studies using human tarsal conjunctival biopsies.

| **Title** | **Study summary** | **Sample size / Participants** | **Location** | **Key findings** | **Comments** |
| --- | --- | --- | --- | --- | --- |
|  |  |  |  |  |  |
| The pathology of trachoma in a black South African population[13] | Descriptive case series of active and scarring trachoma. | 33 cases of varying ages. | South Africa | A mixed inflammatory cell infiltrate, especially in children. Older adults also had a marked infiltrate, mainly of plasma cells. |  |
| Conjunctival lymphocyte subsets in trachoma[14] | Case-control study of adults with TT and (unmatched) controls measuring T cell subtypes, B cells and IgG/IgM/IgA. | 21 cases (11 with inflammation) and 3 controls. | Saudi Arabia | Cases had increased T cells of different subtypes. Inflamed cases had increased B cells. Antibodies were found in all cases (IgM only if inflamed), | No demographic details. |
| Immunopathology of trachomatous conjunctivitis[15] | Descriptive case series of children with active trachoma. | 8 cases. | Egypt | Epithelial hyperplasia and HLA-DR expression. A mixed inflammatory cell infiltrate of the epithelium and stroma. Follicles composed largely of B cells. Plasma cells predominantly IgA, some IgG also. |  |
| T cells and trachoma. Their role in cicatricial disease[16] | Case-control study of adults with TT and (unmatched) controls. | 14 cases (3 with inflammation) and 3 controls. | USA | A chronic inflammatory cell infiltrate was seen in cases, mainly lymphocytes. T cells (consisting of CD4+ and CD8+) outnumbered B cells. | Unclear whether cases and controls comparable - no demographic details; control subjects all had fatal systemic disease. |
| The histopathology and mechanism of entropion in patients with trachoma[17] | Descriptive case series of adults with TT. | 11 cases (without inflammation). | Saudi Arabia | Atrophic epithelium; compact scar tissue with parallel fibres; loss of goblet cells. |  |
| Immunopathogenesis of conjunctival scarring in trachoma[18] | Case-control study of children with active trachoma and controls looking for IL-1α, IL-1β, TNF-α and PDGF. | 9 cases and 4 controls. | Saudi Arabia | Epithelial cells expressed IL-1α and IL-1β. Marcrophages expressed IL-1α, IL-1β, TNF-α and PDGF. No cytokines detected in controls. |  |
| Collagen content and types in trachomatous conjunctivitis[19] &  An immunohisotochemical study of collagens in trachoma and vernal keratoconjunctivitis[20] | Case-control studies of different stages of trachoma analysing collagen types. | 9 children with active disease and 4 controls; 9 adults with scarring and 5 controls. | Saudi Arabia | Active disease showed increased types I, III and IV collagen, and some type V collagen (not seen in controls). Scaring showed marked deposition of types IV and V collagen. | Overlap of patients with previous study.[18] |
| A survey of trachoma: the histopathology and the mechanism of progressive cicatrisation of eyelid tissues[21] | Descriptive case series of different stages of trachoma. | 5 children with active disease; 21 adults with TT. | Turkey | Active disease showed lymphoid follicles with surrounding mixed inflammatory cell infiltrate. Scarring showed epithelial squamous metaplasia/atrophy and collagen scar tissue. |  |
| Expression of gelatinase B (MMP-9) in trachomatous conjunctivitis[22] | Case-control study of children with active trachoma and controls using immunohistochemistry to look for MMP-9 and CD68 and zymography for MMP-9. | 6 active trachoma cases and 7 controls. | Saudi Arabia | MMP-9 found in macrophages in all cases but no controls. Cases also had more MMP-9 with zymography. |  |
| Expression of growth factors in the conjunctiva from patients with active trachoma[23] | Case-control study of children with active trachoma and control children, using mono/poly-clonal antibodies for CTGF, bFGF, VEGF and tenascin. | 6 active trachoma cases and 6 controls | Saudi Arabia | Trachomatous epithelium had upregulated VEGF, and macrophages had increased expression of CTGF and bFGF. Cases also had increased CD105 and tenascin. | Overlap of patients with previous study.[22] |

**Table S3***.* Quantitative gene expression and microarray studies from human tarsal conjunctival swab samples.

| **Title** | **Study summary** | **Sample size / Participants** | **Location** | | **Key findings** | | | **Comments** |
| --- | --- | --- | --- | --- | --- | --- | --- | --- |
|  |  |  |  | |  | | |  |
| Evidence for a predominantly proinflammatory conjunctival cytokine response in individuals with trachoma[24] | Cross-sectional study measuring INF-γ, IL-1β, IL-2, IL-4, IL-5, IL-6, IL-10, IL-12p40, TGF-β1, TNF-α and CD3-δ. | 50 people of varying ages and disease status. | Tanzania |  | | | Infection was associated with upregulated INF-γ, IL-1β, IL-2, TGF-β1 and TNF-α. Active disease was associated with upregulated INF-γ, IL-2, IL-12p40, TGF-β1 and TNF-α. Scarring was associated with upregulated TGF-β1. | β-actin used as housekeeping gene. TGF-β1 is post-transcriptionally regulated and subsequent studies have not found it to be associated with disease. |
| Cytokine and fibrogenic gene expression in the conjunctivas of subjects from a Gambian community where trachoma is endemic[25] | Population based cross-sectional study measuring INF-γ, IL-1β, IL-2, IL-4, IL-10, IL-12p35, IL-12p40, TGF-β2, TNF-α, MMP-1, MMP-9, perforin and Collagen I. | 248 people of varying ages, 42 of whom had active trachoma, 17 had Ct infection. | The Gambia | | | Active disease without *Ct* infection associated with upregulated IL-1β, IL-10, TNF-α, and MMP-9. *Ct* infection (with or without disease) was additionally associated with upregulated INF-γ, IL-12p40, perforin and IL-4. | | No comment made on individuals with scarring. TGF-β1 levels too low to measure. TGF-β2 was constitutively expressed across all clinical categories. |
| Temporal cytokine gene expression patterns in subjects with trachoma identify distinct conjunctival responses associated with infection[26] | Longitudinal study of children, with examination every 2 weeks for 24 weeks including swabs for measuring INF-γ, IL-1β, IL-10, IL-12p40 and TNF-α. | 16 children: at baseline 9 had active disease, 7 were clinically normal. | The Gambia | | | Infection without disease associated with upregulated INF-γ, IL-1β, IL-12p40. Infection with disease associated with greater upregulation of all cytokines. | | Simplified WHO grading system used despite small sample size. |
| Conjunctival FOXP3 expression in trachoma: do regulatory T cells have a role in human ocular chlamydia trachomatis infection?[27] | Cross-sectional study of children measuring INF-γ, IDO, IL-10 and FOXP3. | 345 children: 74 with active disease, 20 with scarring, 251 clinically normal. | The Gambia | | | Infection without disease associated with upregulated INF-γ and IDO. FOXP3 upregulated in disease without infection. Infection with disease was associated with greater upregulation of INF-γ, IDO, IL-10 and FOXP3. | |  |
| Conjunctival expression of MMP and proinflammatory cytokine genes after trichiasis surgery[28] | Longitudinal study measuring IL-1β,TNF-α, MMP-1, MMP-2, MMP-9, TIMP-1, and TIMP-2 a year after TT surgery. | 240 cases | The Gambia | | | Recurrent TT associated with reduced MMP-1/TIMP-1 ratio. Inflammation associated with upregulated IL-1β, TIMP-2 and, if non-*Ct* bacterial infection also present, with TNF-α and MMP-9. | |  |
| Pathway-focused arrays reveal increased MMP-7 transcription in trachomatous trichiasis[29] | Case-control study of adults with TT and controls involving microarray experiments focused on ECM and adhesion molecules and human Th1/Th2/Th3 cells. Confirmatory qPCR gene expression of selected targets performed in an independent case-control study. MMP-7 level was measured. | 11 cases and 11 controls. N=94 for qPCR study. | The Gambia | | | MMP-7 upregulation was the only consistent result between array and qPCR results. No evidence for Th1/Th2/Th3 polarization was found. | |  |
| Human conjunctival transcriptome analysis reveals the prominence of innate defense in *Ct* infection[30] | Case-control study of children with active disease and controls involving transcriptome-wide microarray experiments. Confirmatory qPCR gene expression of selected targets was performed in an independent case-control study. | 20 cases with active disease and infection; 20 cases with active disease without infection; 20 controls. | The Gambia | | | Gene enrichment showed the top-ranking gene ontology terms for disease/infection were typical of immune system activation, epithelial cell integrity, apoptosis, cell death, leukocyte migration and IL-receptor activity. Quantitative PCR results were consistent with the array data. | |  |
| Conjunctival transcriptome in scarring trachoma[31] | Case-control study of adults with TT and control subjects involving transcriptome-wide microarray experiments. Confirmatory qPCR gene expression of selected targets was performed in an independent case-control study. | Microarray analysis: 15 TT without inflammation; 13 TT with inflammation; 14 controls.  qPCR study:  386 TT cases, 386 controls | Ethiopia | | | Gene enrichment results were consistent with squamous metaplasia of the epithelium, an activated innate immune response especially when inflammation was present (IL1B, CXCL5, S100A7), cytoskeletal remodelling (MMP7, MMP9, MMP12), limited Th1 response (INDO, NOS2A), and no evidence for a Th2 response. Quantitative PCR results were consistent with the array data. | |  |
| Active trachoma is associated with increased conjunctival expression of *IL17A* and pro-fibrotic cytokines[32] | Population based cross-sectional study measuring S100A7, IL1B, IL17A, IL23A, CXCL5, CCL18, TLR2, NLRP3, KLRD1, CTGF and MMP9. | 470 chilldren. | Tanzania | | | Active disease was associated with upregulated S100A7, IL17A, CCL18, CXCL5 and CTGF. Non-chlamydial bacterial infection was associated with upregulated IL17A, CXCL5, CCL18 and KLRD1. | |  |
| Innate immune responses and modified extracellular matrix regulation characterise bacterial infection and cellular/connective tissue changes in scarring trachoma[33] | Case-control study of adults with early conjunctival scarring and controls measuring INF-γ, INDO, TNFA, IL-1B, IL-10, IL-12B, IL-13, IL-13RA2, S100A7, DEFB4A, CXCL5, SAA1, ARG1, NOS2, MMP-1, MMP-7, MMP-9, MMP-10, MMP-12, TIMP-1, SPARCL1, CFH and CD83. Infection status was ascertained and *in vivo* confocal microscopy performed. | 363 cases and 363 controls. | Tanzania | | | Scarring was associated with upregulated S100A7, SAA1, DEFB4A, CXCL5, MMP12, INDO, MMP9, IL1B, NOS2, MMP7, CD83, TNFA, TIMP1; and with downregulated SPARCL1, MMP-10 and CFH. Many of these genes were also differentially regulated with the presence of nonchlamydial bacterial infection. | |  |

**Table S4.** Lymphoproliferative and cytokine studies using human PBMCs.

| **Title** | **Study summary** | **Sample size / Participants** | **Location** | **Key findings** | **Comments** |
| --- | --- | --- | --- | --- | --- |
|  |  |  |  |  |  |
| Conjunctival scarring in trachoma is associated with depressed cell-mediated immune responses to chlamydial antigens[34] | Case-control study of cases with scarring and controls, measuring lymphoproliferative responses and INF-γ production in response to *Ct* EB, MOMP and cHsp60. *Ct* LPS was detected by ELISA and *Ct* DNA detected by PCR. | 29 TS cases (uninflamed) and 29 controls | The Gambia | Cases had reduced proliferative responses to all 3 *Ct* antigens, a small reduction in INF-γ secretion and more *Ct* infection. | . |
| Subjects recovering from human ocular chlamydial infection have enhanced lymphoproliferative responses to chlamydial antigens compared with those of persistently diseased controls[35] | Longitudinal study following children with active trachoma for 6 months, examined every 2-4 weeks. Lymphoproliferative responses and INF-γ production to *Ct* EB, MOMP and cHSP60 measured after 4-5 months. *Ct* LPS was detected by IDEIA. | 47 active trachoma cases: 26 had persistent clinical disease throughout the study period; 21 resolved disease. | The Gambia | Children who resolved disease had stronger proliferative responses to all *Ct* antigens. No difference in INF-γ production. Persistent disease was associated with *Ct* infection. |  |
| Th1/Th2 profiles of peripheral blood mononuclear cells; responses to antigens of Ct in subjects with severe trachomatous scarring[36] | Case-control study of cases with scarring and controls, measuring lymphoproliferative responses, INF-γ production and gene expression (INF-γ, IL-4 and IL-10) in response to *Ct* EBs, MOMP and cHsp60. *Ct* LPS was detected by ELISA. | 30 TS cases (uninflamed) and 30 controls. | The Gambia | cHsp60 caused increased IL-4 producing cells in cases and increased INF-γ production in controls. MOMP caused increased INF-γ producing cells in controls. |  |
| Systemic effector and regulatory immune responses to chlamydial antigens in trachomatous trichiasis[37] | Case-control study of cases with scarring and controls, measuring lymphoproliferative responses and production of IFNγ, TNFα, IL5, IL10, IL12p40, and IL13 and also cellular source of IFNγ, IL10, FOXP3, CTLA4, GITR, in response to Ct EBs, MOMP, PmpD, PmpG and Omp2. | 42 TT cases and 42 controls (17 pairs analysed for cellular source; 15 pairs analysed for NK cell markers). | The Gambia | No significant differences in lymphoproliferation, cytokine levels or cell types between cases and controls detected. NK cells identified to be a major, early source of IFNγ, which increased with age. |  |

**Table S5.** Antibody / B cell responses from human serum, conjunctival and tear samples.

| **Title** | **Study summary** | **Sample size / Participants** | **Location** | **Key findings** | **Comments** |
| --- | --- | --- | --- | --- | --- |
|  |  |  |  |  |  |
| Fluorescent antibodies in the fluid of the conjunctival sac of trachoma patients[38] | Case-control study of children with active disease and controls measuring antibodies to TRIC in conjunctival fluid and (in a subset only) serum. | 21 cases and 22 controls. | Israel | 10 cases had antibodies detected compared to none of the controls. Conjunctival titres were higher than serum titres in some patients. | Method of recruitment and demographic details not given. MacCallan grading classification used. |
| Antibodies to trachoma in eye secretions of Saudi Arab children[39] | Case-control study of children measuring antibodies to TRIC in conjunctival fluid and tears. Chlamydial culture and detection of inclusion bodies from conjunctival swabs also performed. | 81 Saudi Arabian children (and 30 Caucasian children. | Saudi Arabia | 50 Saudi Arabian children had antibodies compared to none of the Caucasian children. Saudi Arabian children who were antibody positive were more likely to have active trachoma, to have inclusion bodies seen and to be culture positive. | MacCallan grading classification used. |
| The serum and conjunctival antibody response to trachoma in Gambian children[40] | Longitudinal study measuring IgG and IgA antibodies to TRIC in tears and IgG antibodies in serum, on 6 occasions over 61 weeks. Detection of inclusion bodies from conjunctival swabs also performed. | 99 children, the majority of whom had active disease at some point. | The Gambia | Higher serum antibody titres were strongly associated with clinical disease. Tear IgG was less strongly associated, and IgA even less. Serum IgG titres were higher in diseased children if inclusions were found. | Modified MacCallan grading classification used. |
| Local and humoral chlamydial antibodies in trachoma patients of different age groups[41] | Cross-sectional study measuring antibodies to TRIC in tears and serum in cases with trachoma. | 194 cases of varying ages with different stages of trachoma. | Israel | Average antibody titres in tears tended to decrease with age, and in sera to increase with age. | MacCallan grading classification used. Infection status and comparison with controls not shown, |
| Antichlamydial antibody in tears and sera, and serotypes of Ct isolated from schoolchildren in Sourthern Tunisa[42] | Cross-sectional study measuring antibodies to TRIC in tears and serum. Chlamydial culture was also performed. | 94 school children aged 6-10 years (tears collected in 71 children). | Tunisia | Antibody titres, especially in tears, showed some correlation with level of conjunctival inflammation. Titres were higher if culture positive. |  |
| Conjunctival scarring in trachoma is associated with depressed cell-mediated immune responses to chlamydial antigens[34] | Same study as in section above: serum antibody responses to *Ct* EB, MOMP and cHsp60 also measured. | 29 cases and 29 controls | The Gambia | Scarring was associated with increased IgG and decreased IgA to *Ct* EB. |  |
| The influence of local antichlamydial antibody on the acquisition and persistence of human ocular chlamydial infection: IgG antibodies are not protective[43] | Longitudinal, community-wide study with examinations at 0, 7 and 20 months including conjunctival swabs for *Ct* antibodies and infection. Serum for *Ct* antibody responses taken at baseline and from a subset at 7 months. | 771 without disease at baseline (37 developed disease); 184 with disease at baseline (64 with persistent disease). | The Gambia | High levels of IgG from swab samples associated with an increased risk of developing incident active disease, there was an opposite trend for IgA (p=0.13). Serum antibody responses not significantly different. | Results were adjusted for sharing a room with an active case as a marker of exposure to infection. Unclear whether results refer to risk of incident infection or clinical disease. |
| Characterization of B-cell responses to Ct antigens in humans with trachoma[44] | Case-control study using the ELISPOT assay to measure cells actively secreting antibody to *Ct* EB, MOMP and cHsp60 in the serum. Serum IgG and IgA responses to MOMP also measured. | 41 children with active disease (36 with TF, 5 with TI) and 19 controls; 17 adults with scarring and 17 controls. | The Gambia | Children with TI had an almost absent B cell response of all isotypes to all *Ct* antigens (lower than controls). Children with TF had increased IgA secreting cells. Adults had similar numbers of B cells. Antibody responses not significantly different. | Control children and adults had clearly detectable actively secreting B cells. Infection with Ct not confirmed. |
| Antibody response to the 60-kDa cHsp is associated with scarring trachoma[45] | Case-control study of scarred subjects and controls, measuring serum IgG responses to *Ct* EB and cHsp60. | 148 cases and 148 controls. | The Gambia | Seroprevalence of *Ct* infection around 90% in cases and controls. cHsp60 IgG was detected in more cases than controls (32% vs 16%), even after stratifying for *Ct* titre. | Although more cases than controls were seropositive for cHsp60, the overall proportion positive was relatively low compared to genital tract infection. |
| Immune response to chlamydial 60-kilodalton heat shock protein in tears from Nepali trachoma patients[46] | Cross-sectional study measuring serum and tear antibody responses to cHsp60, MOMP and cHsp60 fusion proteins. Chlamydial antibody titres were measured using MIF to EBs. | 146 people of varying ages: 40 with TF, 53 with TI, 37 with TS, 16 disease-free. | Nepal | Active and scarring disease were associated with tear cHsp60 IgG. Serum cHsp60 IgG was associated with TI only. Almost all individuals were seropositive by MIF. |  |
| Characterization of humoral immune responses to chlamydial Hsp60, CPAF and CT795 in inflammatory and severe trachoma[47] | Case-control study measuring tear antibody responses to cHsp60, CPAF and CT795. | 65 cases with active disease and 65 controls; 59 cases with TT and 59 controls. | Nepal | Active cases had elevated IgG to all antigens (more so when infection was present) and elevated IgA to cHsp60, compared to controls. TT cases had significantly elevated IgG to CPAF and reduced IgA to CT795. |  |

**Table S6.** Miscellaneous human trachoma studies.

| **Title** | **Study summary** | **Sample size / Participants** | **Location** | **Key findings** | | **Comments** |
| --- | --- | --- | --- | --- | --- | --- |
|  |  |  |  |  | |  |
| Expression of MHC class II antigens by conjunctival epithelial cells in trachoma: implications concerning the pathogenesis of blinding disease[48] | Case-control study of children with active disease and controls, assessing MHC class II expression on conjunctival epithelial cells from swab samples. *Ct* antigen was detected by IDEIA. | 40 cases and 38 controls. | The Gambia | | MHC class II expression found and was associated with papillary inflammation. |  |
| Cell-mediated immunity in trachomatous scarring[49] | Comparative study of subjects with leprosy compared to siblings without leprosy, in whom the grade of trachomatous conjunctival scarring was discordant. | 50 sibling pairs involving paucibacillary and 12 involving multibacillary leprosy. | Ethiopia | | Multibacillary leprosy (taken to indicate suppressed cell-mediated immunity) was associated with reduced conjunctival scarring. Paucibacillary leprosy (taken to indicate enhanced cell-mediated immunity) was associated with increased scarring. |  |
| Synthetic peptides based on *Ct* antigens identify cytotoxic T lymphocytes in subjects from a trachoma-endemic population[50] | Case-control study of adults with conjunctival scarring, control adults and children with active trachoma but no infection. CTL responses to synthetic peptides based on MOMP and cHhsp60 were measured in appropriate HLA individuals. | 12 adult cases, 10 adult controls and 4 children. | The Gambia | | CTL responses were found in 6 subjects (3 to MOMP and 3 to cHsp60), all of whom were either adult controls or children. | Method for detecting *Ct* infection not shown. |
| Serum complement components in patients with trachoma[51] | Case-control study measuring serum C1q, C3, C4 and C5 levels. | 98 cases and 56 controls, of varying ages. | India | | Serum C1q and C3 were lower in active disease and returned to normal after treatment. | Controls probably not matched for risk of trachoma. MacCallan grading classification used. |
| Failure to detect HLA-A*6802-restricted T cells specific for Ct antigens in subjects from trachoma-endemic communities[52] | Case-control study of subjects with conjunctival scarring and controls. CD8+ T cell responses were measured to synthetic HLA-A*6802 predicted peptides based on MOMP, MIP and cHsp70. | 10 cases with scarring and 10 controls (children and adults). | The Gambia | | No significant responses were observed. |  |
| The frequency of *Ct* major outer membrane protein-specific CD8+ T lymphocytes in active trachoma is associated with current ocular infection[53] | Longitudinal study with examinations every 2 weeks for 28 weeks. Peptide tetramers to MOMP were used to detect CD8+ cells in appropriate HLA individuals. PCR was used to detect *Ct* DNA. | 86 children, around 20% of whom had infection and/or disease at the outset. | The Gambia | | *Ct*-specific cells were found relatively infrequently, but there was some association with *Ct* infection. Such cells were not associated with disease. |  |
| Role of secreted conjunctival mucosal cytokine and chemokines proteins in different stages of trachomatous disease[54] | Cross-sectional study with matched controls using multiplex bead analysis to measure levels of IFN-α, IFN-γ, IL-1β, IL-1Ra, IL-2, IL-2R, IL-4, IL-5, IL-6, IL-7, IL-8, IL-10, IL-12p40, IL-13, IL-15, IL-17, TNF-α, GM-CSF, CCL2, CCL3, CCL4, CCL5, CCL11, CXCL9 and CXCL10 in tear samples. *Ct* DNA level was measured (with or without PCR). | 208 individuals of varying ages and disease status. | Nepal | | Active disease was associated with increased IL-6, IL-8, IL-10, TNF-α and CCL-2. Scarring was associated with increased IL-1β, IL-2, IL-6, IL-8, IL-10, IL-15, TNF-α, CCL-2, CCL-11 and CXCL-2; and with reduced IL-1Ra, IL-4, IL-12p40 and IL-13. Infection in cases was associated with increased IL-6, IL-10, IL-15, TNF-α, CCL-4 and CCL-5. | Selection of subjects unclear. Further significant associations are presented for clinical subgroups, but p-values are unadjusted. |

**References**

1. Conway DJ, Holland MJ, Campbell AE, Bailey RL, Krausa P, et al. (1996) HLA class I and II polymorphisms and trachomatous scarring in a Chlamydia trachomatis-endemic population. J Infect Dis 174: 643-646.

2. White AG, Bogh J, Leheny W, Kuchipudi P, Varghese M, et al. (1997) HLA antigens in Omanis with blinding trachoma: markers for disease susceptibility and resistance. Br J Ophthalmol 81: 431-434.

3. Conway DJ, Holland MJ, Bailey RL, Campbell AE, Mahdi OS, et al. (1997) Scarring trachoma is associated with polymorphism in the tumor necrosis factor alpha (TNF-alpha) gene promoter and with elevated TNF-alpha levels in tear fluid. Infect Immun 65: 1003-1006.

4. Mozzato-Chamay N, Mahdi OS, Jallow O, Mabey DC, Bailey RL, et al. (2000) Polymorphisms in candidate genes and risk of scarring trachoma in a Chlamydia trachomatis--endemic population. J Infect Dis 182: 1545-1548.

5. Natividad A, Wilson J, Koch O, Holland MJ, Rockett K, et al. (2005) Risk of trachomatous scarring and trichiasis in Gambians varies with SNP haplotypes at the interferon-gamma and interleukin-10 loci. Genes Immun 6: 332-340.

6. Natividad A, Cooke G, Holland MJ, Burton MJ, Joof HM, et al. (2006) A coding polymorphism in matrix metalloproteinase 9 reduces risk of scarring sequelae of ocular Chlamydia trachomatis infection. BMC Med Genet 7: 40.

7. Natividad A, Hanchard N, Holland MJ, Mahdi OS, Diakite M, et al. (2007) Genetic variation at the TNF locus and the risk of severe sequelae of ocular Chlamydia trachomatis infection in Gambians. Genes Immun 8: 288-295.

8. Natividad A, Holland MJ, Rockett KA, Forton J, Faal N, et al. (2008) Susceptibility to sequelae of human ocular chlamydial infection associated with allelic variation in IL10 cis-regulation. Hum Mol Genet 17: 323-329.

9. Atik B, Skwor TA, Kandel RP, Sharma B, Adhikari HK, et al. (2008) Identification of novel single nucleotide polymorphisms in inflammatory genes as risk factors associated with trachomatous trichiasis. PLoS ONE 3: e3600.

10. Abbas M, Bobo LD, Hsieh YH, Berka N, Dunston G, et al. (2009) Human leukocyte antigen (HLA)-B, DRB1, and DQB1 allotypes associated with disease and protection of trachoma endemic villagers. Invest Ophthalmol Vis Sci 50: 1734-1738.

11. Natividad A, Hull J, Luoni G, Holland M, Rockett K, et al. (2009) Innate immunity in ocular Chlamydia trachomatis infection: contribution of IL8 and CSF2 gene variants to risk of trachomatous scarring in Gambians. BMC Med Genet 10: 138.

12. Bailey RL, Natividad-Sancho A, Fowler A, Peeling RW, Mabey DC, et al. (2009) Host genetic contribution to the cellular immune response to Chlamydia trachomatis: Heritability estimate from a Gambian twin study. Drugs Today (Barc) 45 Suppl B: 45-50.

13. Abrahams C, Ballard RC, Sutter EE (1979) The pathology of trachoma in a black South African population. Light microscopical, histochemical and electron microscopical findings. S Afr Med J 55: 1115-1118.

14. Burd EM, Tabbara KF, Nasr AM, Taylor PB (1988) Conjunctival lymphocyte subsets in trachoma. Int Ophthalmol 12: 53-57.

15. el-Asrar AM, Van den Oord JJ, Geboes K, Missotten L, Emarah MH, et al. (1989) Immunopathology of trachomatous conjunctivitis. Br J Ophthalmol 73: 276-282.

16. Reacher MH, Pe'er J, Rapoza PA, Whittum-Hudson JA, Taylor HR (1991) T cells and trachoma. Their role in cicatricial disease. Ophthalmology 98: 334-341.

17. al-Rajhi AA, Hidayat A, Nasr A, al-Faran M (1993) The histopathology and the mechanism of entropion in patients with trachoma. Ophthalmology 100: 1293-1296.

18. Abu el-Asrar AM, Geboes K, Tabbara KF, al-Kharashi SA, Missotten L, et al. (1998) Immunopathogenesis of conjunctival scarring in trachoma. Eye 12 ( Pt 3a): 453-460.

19. Abu el-Asrar AM, Geboes K, al-Kharashi SA, Tabbara KF, Missotten L (1998) Collagen content and types in trachomatous conjunctivitis. Eye 12 ( Pt 4): 735-739.

20. Abu el-Asrar AM, Geboes K, al-Kharashi SA, al-Mosallam AA, Tabbara KF, et al. (1998) An immunohistochemical study of collagens in trachoma and vernal keratoconjunctivitis. Eye 12 ( Pt 6): 1001-1006.

21. Guzey M, Ozardali I, Basar E, Aslan G, Satici A, et al. (2000) A survey of trachoma: the histopathology and the mechanism of progressive cicatrization of eyelid tissues. Ophthalmologica 214: 277-284.

22. El-Asrar AM, Geboes K, Al-Kharashi SA, Al-Mosallam AA, Missotten L, et al. (2000) Expression of gelatinase B in trachomatous conjunctivitis. Br J Ophthalmol 84: 85-91.

23. Abu El-Asrar AM, Al-Kharashi SA, Missotten L, Geboes K (2006) Expression of growth factors in the conjunctiva from patients with active trachoma. Eye 20: 362-369.

24. Bobo L, Novak N, Mkocha H, Vitale S, West S, et al. (1996) Evidence for a predominant proinflammatory conjunctival cytokine response in individuals with trachoma. Infect Immun 64: 3273-3279.

25. Burton MJ, Bailey RL, Jeffries D, Mabey DC, Holland MJ (2004) Cytokine and fibrogenic gene expression in the conjunctivas of subjects from a Gambian community where trachoma is endemic. Infect Immun 72: 7352-7356.

26. Faal N, Bailey RL, Sarr I, Joof H, Mabey DC, et al. (2005) Temporal cytokine gene expression patterns in subjects with trachoma identify distinct conjunctival responses associated with infection. Clin Exp Immunol 142: 347-353.

27. Faal N, Bailey RL, Jeffries D, Joof H, Sarr I, et al. (2006) Conjunctival FOXP3 expression in trachoma: do regulatory T cells have a role in human ocular Chlamydia trachomatis infection? PLoS Med 3: e266.

28. Burton MJ, Bailey RL, Jeffries D, Rajak SN, Adegbola RA, et al. (2010) Conjunctival expression of matrix metalloproteinase and proinflammatory cytokine genes after trichiasis surgery. Invest Ophthalmol Vis Sci 51: 3583-3590.

29. Holland MJ, Jeffries D, Pattison M, Korr G, Gall A, et al. Pathway-focused arrays reveal increased matrix metalloproteinase-7 (matrilysin) transcription in trachomatous trichiasis. Invest Ophthalmol Vis Sci 51: 3893-3902.

30. Natividad A, Freeman TC, Jeffries D, Burton MJ, Mabey DC, et al. (2010) Human conjunctival transcriptome analysis reveals the prominence of innate defense in Chlamydia trachomatis infection. Infect Immun 78: 4895-4911.

31. Burton MJ, Rajak SN, Bauer J, Weiss HA, Tolbert SB, et al. (2011) Conjunctival transcriptome in scarring trachoma. Infect Immun 79: 499-511.

32. Burton MJ, Ramadhani A, Weiss HA, Hu V, Massae P, et al. (2011) Active Trachoma Is Associated with Increased Conjunctival Expression of IL17A and Profibrotic Cytokines. Infect Immun 79: 4977-4983.

33. Hu VH, Weiss HA, Ramadhani AM, Tolbert SB, Massae P, et al. (2012) Innate immune responses and modified extracellular matrix regulation characterize bacterial infection and cellular/connective tissue changes in scarring trachoma. Infect Immun 80: 121-130.

34. Holland MJ, Bailey RL, Hayes LJ, Whittle HC, Mabey DC (1993) Conjunctival scarring in trachoma is associated with depressed cell-mediated immune responses to chlamydial antigens. J Infect Dis 168: 1528-1531.

35. Bailey RL, Holland MJ, Whittle HC, Mabey DC (1995) Subjects recovering from human ocular chlamydial infection have enhanced lymphoproliferative responses to chlamydial antigens compared with those of persistently diseased controls. Infect Immun 63: 389-392.

36. Holland MJ, Bailey RL, Conway DJ, Culley F, Miranpuri G, et al. (1996) T helper type-1 (Th1)/Th2 profiles of peripheral blood mononuclear cells (PBMC); responses to antigens of Chlamydia trachomatis in subjects with severe trachomatous scarring. Clin Exp Immunol 105: 429-435.

37. Gall A, Horowitz A, Joof H, Natividad A, Tetteh K, et al. (2011) Systemic effector and regulatory immune responses to chlamydial antigens in trachomatous trichiasis. Front Microbiol 2: 10.

38. Bernkopf H, Orfila J, Maythar B (1966) Fluorescent antibodies in the fluid of the conjunctival sac of trachoma patients. Nature 209: 725-726.

39. McComb DE, Nichols RL (1969) Antibodies to trachoma in eye secretions of Saudi Arab children. Am J Epidemiol 90: 278-284.

40. Collier LH, Sowa J, Sowa S (1972) The serum and conjunctival antibody response to trachoma in Gambian children. J Hyg (Lond) 70: 727-740.

41. Maythar B, Zakay-Rones Z (1972) Local and humoral chlamydial antibodies in trachoma patients of different age groups. Invest Ophthalmol 11: 651-654.

42. Treharne JD, Dwyer RS, Darougar S, Jones BR, Daghfous T (1978) Antichlamydial antibody in tears and sera, and serotypes of Chlamydia trachomatis isolated from schoolchildren in Southern Tunisia. Br J Ophthalmol 62: 509-515.

43. Bailey RL, Kajbaf M, Whittle HC, Ward ME, Mabey DC (1993) The influence of local antichlamydial antibody on the acquisition and persistence of human ocular chlamydial infection: IgG antibodies are not protective. Epidemiol Infect 111: 315-324.

44. Ghaem-Maghami S, Bailey RL, Mabey DC, Hay PE, Mahdi OS, et al. (1997) Characterization of B-cell responses to Chlamydia trachomatis antigens in humans with trachoma. Infect Immun 65: 4958-4964.

45. Peeling RW, Bailey RL, Conway DJ, Holland MJ, Campbell AE, et al. (1998) Antibody response to the 60-kDa chlamydial heat-shock protein is associated with scarring trachoma. J Infect Dis 177: 256-259.

46. Hessel T, Dhital SP, Plank R, Dean D (2001) Immune response to chlamydial 60-kilodalton heat shock protein in tears from Nepali trachoma patients. Infect Immun 69: 4996-5000.

47. Skwor T, Kandel RP, Basravi S, Khan A, Sharma B, et al. Characterization of humoral immune responses to chlamydial heat shock protein 60, protease-like activity factor and hypothetical protein CT795 in inflammatory and severe trachoma. Invest Ophthalmol Vis Sci.

48. Mabey DC, Bailey RL, Dunn D, Jones D, Williams JH, et al. (1991) Expression of MHC class II antigens by conjunctival epithelial cells in trachoma: implications concerning the pathogenesis of blinding disease. J Clin Pathol 44: 285-289.

49. Courtright P, Lewallen S, Howe R (1993) Cell-mediated immunity in trachomatous scarring. Evidence from a leprosy population. Ophthalmology 100: 98-104.

50. Holland MJ, Conway DJ, Blanchard TJ, Mahdi OM, Bailey RL, et al. (1997) Synthetic peptides based on Chlamydia trachomatis antigens identify cytotoxic T lymphocyte responses in subjects from a trachoma-endemic population. Clin Exp Immunol 107: 44-49.

51. Sen DK, Sarin GS, Hiranandani M, Baveja UK (1985) Serum complement components in patients with trachoma. Br J Ophthalmol 69: 707-710.

52. Mahdi OS, Whittle HC, Joof H, Mabey DC, Bailey RL (2001) Failure to detect HLA-A*6802-restricted CD8+ T cells specific for Chlamydia trachomatis antigens in subjects from trachoma-endemic communities. Clin Exp Immunol 123: 68-72.

53. Holland MJ, Faal N, Sarr I, Joof H, Laye M, et al. (2006) The frequency of Chlamydia trachomatis major outer membrane protein-specific CD8+ T lymphocytes in active trachoma is associated with current ocular infection. Infect Immun 74: 1565-1572.

54. Skwor TA, Atik B, Kandel RP, Adhikari HK, Sharma B, et al. (2008) Role of secreted conjunctival mucosal cytokine and chemokine proteins in different stages of trachomatous disease. PLoS Negl Trop Dis 2: e264.
